# Functional activation of insula and dorsal anterior cingulate for conflict control against larger monetary loss in young adults with subthreshold depression: a preliminary study

**DOI:** 10.1038/s41598-022-10989-0

**Published:** 2022-04-28

**Authors:** Je-Yeon Yun, Yoonji Irene Lee, Susan Park, Jong Moon Choi, Soo-Hee Choi, Joon Hwan Jang

**Affiliations:** 1 Seoul National University Hospital, Seoul, Republic of Korea; 2 Yeongeon Student Support Center, Seoul National University College of Medicine, Seoul, Republic of Korea; 3 Department of Psychiatry, Seoul National University Hospital, Seoul, Republic of Korea; 4 Department of Psychology, Louisiana State University, Baton Rouge, USA; 5 Department of Psychiatry, Seoul National University College of Medicine, Seoul, Republic of Korea; 6 Department of Psychiatry, Seoul National University Health Service Center, Gwanak-ro 1, Gwanak-gu, Seoul, 08826 Republic of Korea; 7 Department of Human Systems Medicine, Seoul National University College of Medicine, Seoul, Republic of Korea

**Keywords:** Neuroscience, Psychology, Diseases, Medical research, Neurology

## Abstract

Subthreshold depression (StD) is associated with higher risk of later developing major depressive disorder (MDD). Deficits of goal-directed behaviors regarding the motional, motivational, and conflict control are found in MDD. The current study examined neural underpinning of conflict control against monetary punishment in StD compared to MDD and healthy controls (HC). Seventy-one participants (HC, n = 27; StD, n = 21; MDD, n = 23) in their mid-20’s completed self-reports. Preprocessing of functional magnetic resonance imaging acquired for the Simon task against larger or smaller monetary punishment was conducted using ENIGMA HALFpipe version 1.2.1. Neural correlates of conflict control against monetary punishment that could vary with either diagnosis or PHQ-9 total score were examined using a general linear model of FSL. Simon effect was effective for reaction time and accuracy in every subgroup of diagnosis and regardless of the size of monetary punishment. Conflict control against larger monetary loss was associated with higher functional activation of left insula in StD than HC and MDD. StD showed lower functional activation of left dorsal anterior cingulate (dACC) than MDD for conflict control against larger monetary loss. For conflict control against smaller monetary loss, StD demonstrated higher functional activation of left paracentral lobule and right putamen compared to HC. Directed acyclic graphs showed directional associations from suicidal ideation, sadness, and concentration difficulty to functional activation of paracentral lobule, ventromedial prefrontal cortex (vmPFC), and thalamus for conflict control against monetary loss. Differential functional activation of insula and dACC for conflict control against larger monetary loss could be a brain phenotype of StD. Item-level depressive symptoms of suicidal ideation, sadness, and concentration difficulty could be reflected in the conflict control-related functional activation of paracentral lobule (against smaller monetary loss), vmPFC and thalamus (against larger monetary loss), respectively.

## Introduction

Subthreshold depression (StD) is defined as suffer of two to four depressive symptoms listed in the DSM-5 diagnostic criteria of major depressive disorder (MDD) that must include either depressive mood or anhedonia for at least recent 2 weeks^1^. The estimated prevalence of StD ranges from 5 to 25%^2^. Although presence of anxiety symptoms are associated not with future depression but with future prevalence of anxiety disorder^3^, comorbid anxiety disorder in moderate to severe depression is associated with less reduction of depressive symptoms in response to the pharmacotherapy with antidepressants^4^. Both StD and MDD show family histories of affective disorder, patterns of functional impairment and adverse outcomes. Of note, anhedonia or hyposensitivity to rewards shows positive genetic correlations (in terms of the heritability estimate of single nucleotide polymorphisms) with MDD, schizophrenia, and bipolar disorder^5^. StD is a possible risk factor for poor social and role functioning among the help-seeking youth with anxiety and depressive symptoms^6^ and has been associated with increased risk of future MDD, dysthymia, social phobia, or generalized anxiety disorder^1,7–10^. Conversely, lower level of social support is a possible predictor of conversion to MDD for StD as found in a longitudinal cohort study, in which 44.9% of StD were recovered and 14.7% of MDS converted to MDD within the follow-up periods of maximum 17 years^11^. In addition, StD is one of the risk factor for suicide ideation after exposure to stressors^12^.

Recent brain magnetic resonance imaging (MRI) studies have shown differences of brain regional grey matter volumes, surface curvature, and inter-regional structural–functional connections between StD and healthy controls (HC)^13^. First, regional gray matter volume of orbitofrontal cortex, left temporal gyrus, bilateral globus pallidus, and precentral gyrus are smaller in StD compared to HC^14,15^. Of note, regardless of morbidity for psychiatric disorder(s), reports of anhedonia are associated with smaller volume of orbitofrontal cortex^5^. On the contrary, regional gray matter volumes of left thalamus and right rostral anterior cingulate-medial prefrontal cortices are larger in StD compared to HC^14,15^. Second, StD is associated with weaker structural integrity of brain white matter bundles including the corpus callosum (that connects bilateral cerebral hemispheres), inferior longitudinal (that mediates temporal-occipital regions to anterior temporal areas) and superior longitudinal (via which posterior parietal cortices are linked with frontal regions) fasciculi^16^. Third, associations between the StD and outward curvature of subcortical regions including the hippocampus are found in adolescents^17^. Fourth, strengths of resting state functional connectivity between the left amygdala versus bilateral middle frontal cortices and insula are weaker in StD compared to HC^18^. In short, StD is associated with structural and functional changes in frontal and medial temporal regions, as well as white matter disruptions.

Moreover, structural and functional alterations of fronto-temporal regions and white matter-based brain structural connectivity could possibly mediate conversion from StD to MDD. According to the result of a recent meta-analysis (16 studies, n = 67,318), StD have 1.95 times the rate of developing MDD compared to community-dwelling HC or HC at primary-care setting^1^. Brain-based intermediate marker of StD regarding the prognosis of later developing or non-developing a full-blown MDD index episode could be found in the pattern of cortical development and white matter-based structural connections during adolescence. Expansion of cortical surface area until late adolescence at the orbitofrontal and anterior cingulate cortices are larger for StD subgroups with sustained or later progressing depressive symptoms, compared with other StD subgroups who exhibit earlier reduction of depressive symptoms^19^. In addition, a lower structural integrity (measured using the fractional anisotropy value) of white matter bundle spanning from the anterior body of the corpus callosum to the anterior cingulate cortex in adolescent StD is a risk factor for developing MDD by the 2-year follow-up^13^. Possible risk factors of developing a full-blown MDD are recurrent short episodes of depressive symptoms, lifetime prevalence of comorbid anxiety or substance use disorders, suffer of suicidal ideation, lowered social support, comorbid chronic physical disease, and lowered mental or physical functioning^20^. Also, lower recognition thresholds for facial emotion of sadness is a risk factor of non-remitted depressive symptoms in MDS.

Not only the negative valence of depressive and anxiety symptoms, but also the hypersensitivity to punishments, loss of motivation, and reduced goal-directed behavior have been considered as cardinal features of depression^21,22^. Both StD and MDD have been associated with deficits of cognitive control of goal-directed behavior including the goal selection, response selection (inhibition or suppression), and performance monitoring^23^. For reinforcement learning to optimize the performance after feedbacks of punishment, changed functional activations in brain circuits comprised of insula, thalamus, and habenula are found^24,25^. Differential levels of monetary reward or punishment do not affect the patterns of regional brain functional activation during cognitive control of set shifting^26,27^. On the contrary, task performance against punishment of monetary loss is associated with increased functional activation of left insula, putamen, and bilateral dorsal anterior cingulate, compared to the context of not considering the possibility of monetary punishment^28^. Also, emotional recognition and emotional regulation in response to the monetary reward or punishment are associated with regional functional brain activation of orbitofrontal cortex, anterior cingulate cortex, insula, and amygdala^29–32^. As cognitive behavioral therapy including the cognitive restructuring and problem solving is effective in reducing depressive symptoms for StD^33,34^, better understating of negative valence-cognitive system interaction by examining of neural correlates for cognitive control against the distress of monetary punishment could be helpful in treatment planning of StD.

Collectively, StD is related to the increased risk of later developing major depressive episode, shares deficits of cognitive control with MDD, and shows differential characteristics of brain morphology and brain connectome compared to HC. On the contrary, whether StD could be considered as on the continuum with MDD or not is inconclusive yet, as people with subthreshold (StD) and supra-threshold (MDD) depressive symptoms are distinguished in the longitudinal follow-up^35^ and StD demonstrate heterogeneous prognosis (symptom remission, sustained depressive symptoms, or conversion into MDD)^1^. Accordingly, the current study examined differential neural correlates of conflict control against monetary punishment in StD compared to MDD and HC. Notably, functional neuroimaging studies in which tasks of conflict control (selective attention to the goal-relevant color information and response inhibition for goal-irrelevant spatial information) against monetary punishment for error or delayed responses can be useful for deciphering neural mechanism of negative valence(hypersensitivity to punishments)-cognitive system(cognitive control) interaction in depression^21,22^. We hypothesized that differential functional activation patterns for incongruent trial against larger monetary loss (compared to congruence trial and smaller monetary loss) at brain regions such as insula^24,25,28–32^, dorsal anterior cingulate^28–32^, putamen^28^, thalamus^24,25^, and orbitofrontal cortex^29–32^ in StD compared to HC and/or MDD. Further, considering the possibility that relationships between brain phenotype versus depressive symptom could vary for different dimensions of depressive symptoms (as reported for cortical thickness asymmetry in StD)^36^, we modeled conditional independence among the item-level depressive symptom of Patient Health Questionnaire-9 (PHQ-9)^37,38^ and z-stats of functional activation clusters extracted from the fMRI data for conflict control against monetary punishment.

## Material and methods

### Participants and clinical assessment

A total of 71 undergraduate or graduate students (HC, n = 27; MDD, n = 23; StD, n = 21) participated in the current study. Subjects were recruited during the annual health examination program (for MDD and StD) or using advertisements for college students (for HC) at Seoul National University, Seoul, Republic of Korea. All participants satisfied the following inclusion criteria: (1) 18–35 years of age; (2) no lifetime diagnosis of psychotic disorder, substance use disorder, or loss of consciousness due to head injury; and (3) no use of psychotropic medication within 8 weeks of study participation. Diagnosis of psychiatric disorders (either MDD or StD) or exclusion of lifetime history or current morbidity of psychiatric disorders (for HC) were made based on semi-structured interviews using the MINI-International Neuropsychiatric Interview^39,40^ and clinical decision by licensed psychiatrists. At the time of study, all MDD and StD who participated in the current study experienced one or both of the following symptoms: (1) depressive mood or (2) loss of interest or pleasure over the last 2 weeks. Also, participants also satisfied ≥ 5 (diagnosed as MDD, n = 23) or two to four (classified as StD, n = 21) of the components of item A of the DSM-5 diagnostic criteria for MDD.

All participants completed the following self-reporting measures: First, depressive symptoms and anxiety were reported using the PHQ-9^37,38^, the General Anxiety Disorder-7 (GAD-7)^41,42^, and the State-Trait Anxiety Inventory-State Anxiety (STAI-S)^43,44^. Second, cognitive self-appraisals^45,46^ regarding overall self-worth and stress resilience of regulating emotion, solving problem, and getting social support were measured using the Rosenberg Self Esteem Scale (RSES)^47,48^ and the Resilience Appraisal Scale (RAS)^49,50^. Third, self-referential thoughts about the world in terms of social supports perceived as empathic, reliable, and practical (or not)^51^ were evaluated using the Social Support Scale (SS)^52^. Fourth, negative prospect for one’s future^53^ including the feelings about future, loss of motivation, and future expectations were evaluated using the Beck Hopelessness Scale (BHS)^54,55^. Fifth, the Barratt Impulsiveness Scale (BIS)^56,57^ was applied to measure the impulsivity associated with depressive symptoms in the presence of sub-threshold bipolarity or cyclothymic temperament^58^. Last, life satisfaction for domains of physical, psychological, social and environments were reported using the World Health Organization Quality of Life Abbreviated Version (QOL)^59,60^. This study was approved by the Institutional Review Board of Seoul National University College of Medicine and Hospital (Seoul, Republic of Korea; No. 1608–079-785), and has therefore been performed in accordance with the ethical standards in the 1975 Declaration of Helsinki and its later amendments in 2013. All participants provided written informed consent prior to participation.

### Experimental design

**Figure 1:**
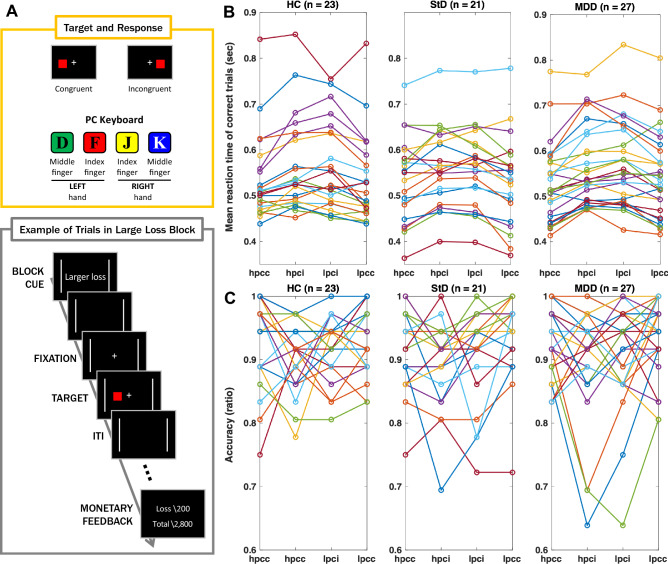
Conflict control against larger or smaller monetary punishment (per error response). (**A**) Task diagram. (**B**) Spaghetti plot of mean reaction times averaged for correct trials per participant (sec). A statistically significant simple two-way interaction between the size of monetary loss versus congruency was found in MDD [F (1, 23) = 4.489, P = 0.045] but not in the HC (P = 0.674) or StD (P = 0.645). For the simple main effects, statistical significance was found for the (in)congruence of target-response location [F (1, 58) = 58.655, P < 0.001) but not for the size of monetary loss (P = 0.653) nor diagnosis (P = 0.649). (**C**) Spaghetti plots of accuracy ratio. Statistically significant simple two-way interactions between the size of monetary loss and congruency were not observed in MDD (P = 0.617), StD (P = 0.294), and HC (P = 0.760). Regarding main effects, statistical significance was found for conflict in target-response location [F (1, 58) = 11.548, P = 0.001] but not for the size of monetary loss (P = 0.068) or diagnosis (P = 0.534). *HC* healthy controls, *StD* subthreshold depression, *MDD* major depressive disorder, *hpci* incongruent trial against larger monetary loss, *hpcc* congruent trial against larger monetary loss, *lpci* incongruent trial against smaller monetary loss, *lpcc* congruent trial against smaller monetary loss.

Each participants underwent three consecutive fMRI scanning sessions. During the fMRI data acquisition, participants underwent a variant of the Simon task^61,62^ (Fig. 1A). Each fMRI session was comprised of 6 blocks, including 3 small monetary punishment blocks and 3 large monetary punishment blocks (per each incorrect or delayed response), pseudo-randomly ordered within each session. Each block consisted of nine trials (congruent or incongruent). As first trials in each block were excluded from the analyses, a total of 144 trials (72 congruent trials and 72 incongruent trials) gathered per participant across the three fMRI sessions were used for the statistical analyses of the Simon task performance.

Each block started with a block cue for 1000 ms followed by a 1000-ms blank screen. At the beginning of each trial, the fixation point was presented for 500 ms followed by a target display. In the target display, a square filled in a target color was presented to the left or right of the fixation point. Participants were instructed to press the “d” key to the green target square with their left middle finger, the “f” key to the red one with their left index finger, the “j” key to the yellow one with their right index finger, and the “k” key to the blue one with their right middle finger before the target disappearance. The red or yellow target was presented in odd trials of each block and the green or blue target was in even trials to avoid repetition. Participants had to respond as quickly as possible to the target stimulus by pressing a button of the same color. The target duration was adjusted dynamically during the practice session by using a staircase procedure, which was applied independently to the trial types (congruent or incongruent) but was identical for both small and large monetary punishment blocks. After nine trials under the same cue for monetary loss (small or large loss) were completed, participants received feedback presented on the screen regarding the amount of monetary loss accrued during the current block and the total monetary balance remaining. Visual stimuli were programmed using Psychtoolbox and responses were collected with a button-box device.

### MRI data acquisition and preprocessing

Brain MRI data were obtained using a Siemens Trio 3.0-Tesla MRI scanner (Siemens Magnetom Trio, Erlangen, Germany) with a 12-channel head coil. Brain fMRI data were acquired during task performance using a T2*-weighted echo-planar imaging BOLD sequence with the following parameters: repetition time (TR) = 2,000 ms, echo time (TE) = 30 ms, flip angle (FA) = 80°, field of view (FOV) = 220 mm, matrix = 64 × 64, 34 contiguous 3.4-mm interleaved axial slices, and voxel size = 3.44 mm × 3.44 mm × 3.40 mm. To achieve magnet-steady images, the first six volumes of each run were discarded. In addition, to detect possible brain pathology and for use in the normalization procedures of fMRI images, high-resolution T1-weighted structural brain MRI scans were obtained using the following parameters: TR = 1,670 ms, TE = 1.89 ms, FA = 9°, FOV = 250 mm, matrix = 256 × 256, number of slices = 208, slice thickness = 1 mm, and voxel size = 0.98 mm × 0.98 mm × 1.00 mm.

Preprocessing of anatomical and functional MRI data were performed using ENIGMA HALFpipe software^63^ version 1.2.1 (http://enigma.ini.usc.edu/protocols/functional-protocols/) that implements fMRIPrep^64,65^. For preprocessing of anatomical T1WI, the T1w image was skull-stripped with antsBrainExtraction.sh (from ANTs^66^) using OASIS (Open Access Series of Imaging Studies) as a target template. Spatial normalization to the ICBM 152 Nonlinear Asymmetrical template^67^ version 2009c (‘MNI152NLin2009cAsym’) was performed through nonlinear registration with antsRegistration^68^ (from ANTs^66^), using brain-extracted versions of both T1w volume and template. Brain tissue segmentation of cerebrospinal fluid (CSF), white matter (WM) and grey matter (GM) was performed on the brain-extracted T1w with FAST^69^ (from FSL^70^).

Preprocessing of the task-based fMRI data were conducted using following procedures^63–65^. First, a reference volume and its skull-stripped version were generated. Second, head-motion parameters with respect to the BOLD reference (transformation matrices and six corresponding rotation and translation parameters) were estimated before any spatiotemporal filtering with MCFLIRT^71^ (Motion-Correction FMRIB’s Linear Image Registration Tool, from FSL^70^). Third, after slice-timing correction, co-registration (six degrees of freedom) of BOLD images to the T1w reference were calculated with bbregister (from FreeSurfer^72^), which implements boundary-based registration^73^. Fourth, the BOLD time-series were resampled onto their original, native space by applying a single, composite transform to correct for head-motion. Fifth, the BOLD time-series were resampled to MNI152NLin2009cAsym standard space, generating a preprocessed, spatially normalized BOLD run. Sixth, grand mean scaling (to set the within-scan mean across all voxels and time-points to a predefined value of 10,000) and spatial smoothing by way of 3dBlurInMask (from AFNI^74,75^) with a Gaussian kernel of FWHM = 6 mm were applied. Seventh, classification of noise component based on ICA-AROMA^76^ and denoising of estimated motion artifacts using fsl_regfilt (from FSL^70^) were performed. Eighth, temporal filtering to remove low-frequency drift via a high-pass was conducted using the Gaussian-weighted temporal filter with 125 s FWHM (from FEAT of FSL^70^). Finally, confounding timeseries of three region-wise global signals extracted within the CSF, the WM and the whole-brain masks were removed from voxel-wise time series via linear regression^77^.

### fMRI BOLD-level analyses: subject-level and group-level analyses

Analysis was performed with the general linear model (GLM) implemented in FEAT (FMRIB’s Expert Analysis Tool). A first-level GLM was run for event-related designs. Five events including the incongruent (hpci) or congruent (hpcc) trials against larger monetary loss, incongruent (lpci) or congruent (lpcc) trials against larger monetary loss, and trials with error response (err; event with no interest) were included as GLM regressors, and were convolved with a double Gamma canonical hemodynamic response function^65^.

**Figure 2:**
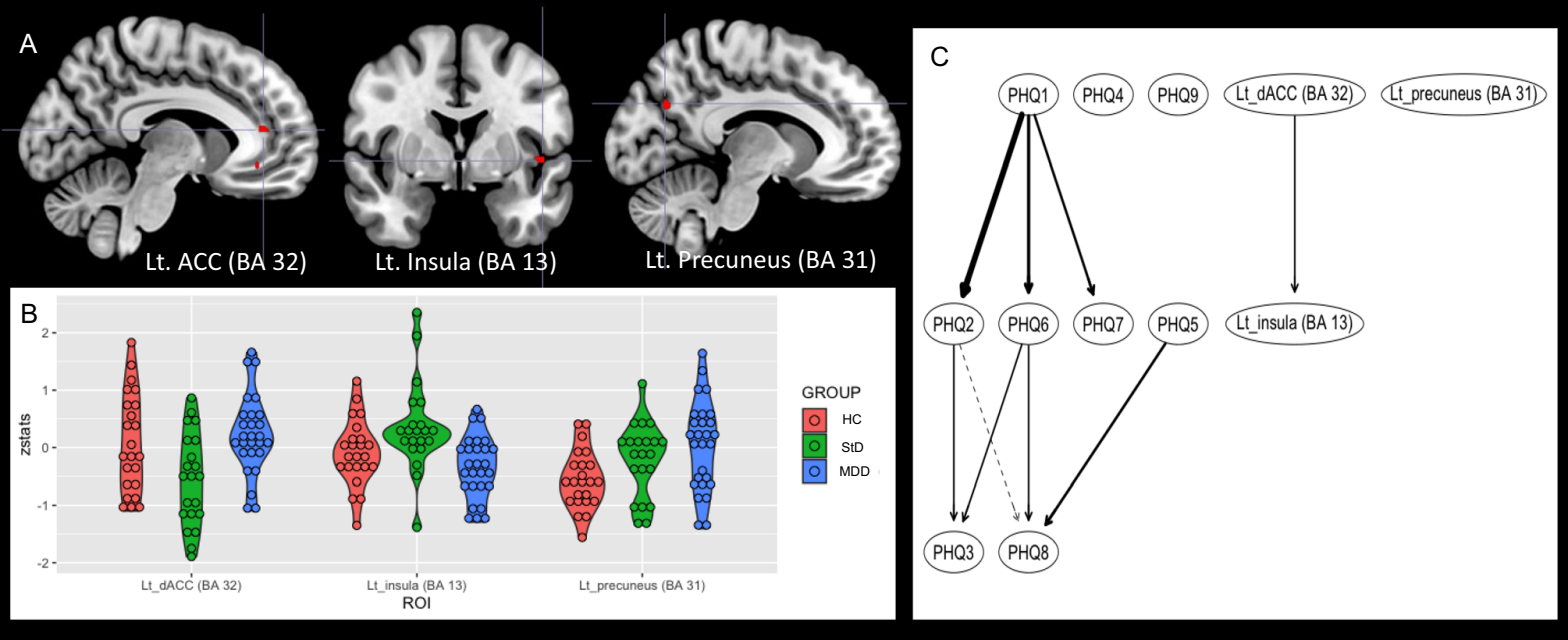
Between-group differences of functional brain activation for conflict control of incongruence trial against larger monetary loss, compared to the congruence trial of target-response location. Conflict control against larger monetary loss was associated with higher functional activation of insula in subthreshold depression (StD) than healthy control (HC) and major depressive disorder (MDD). StD also showed lower functional activation of left dorsal anterior cingulate (dACC) compared to MDD. Higher functional activation of precuneus in MDD compared to HC was also shown. (**A**) Clusters of regional brain functional activation with significant between-group differences (cluster-forming thresholds of P < 0.001 and k ≥ 10). (**B**) Violin plots of z-stats per clusters per group. (**C**) Directed acyclic graph that represents conditional independence associations among depressive symptoms [nine items of Patient-Health Questionnaire-9 (PHQ-9)] and z-stats of 3 brain clusters. *PHQ-1* sadness, *PHQ2* anhedonia, *PHQ3* sleep disturbance, *PHQ4* appetite change, *PHQ5* psychomotor change, *PHQ6* fatigue, *PHQ7* self-reproach, *PHQ8* concentration difficulty, *PHQ9* suicidal ideation, *Lt* left, *BA* Brodmann area.

**Figure 3:**
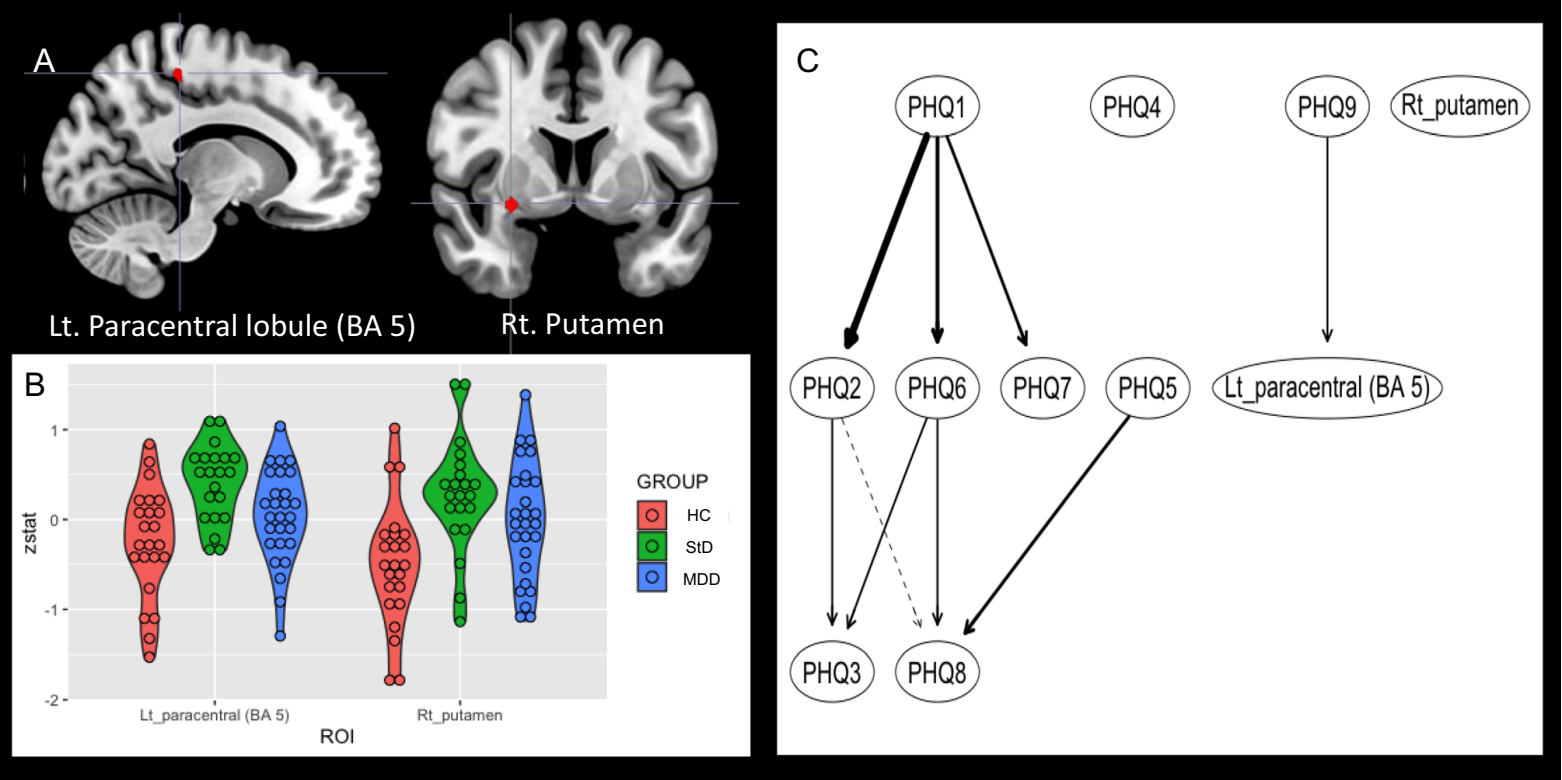
Between-group differences of functional brain activation for conflict control of incongruence trial against smaller monetary loss, compared to the congruence trial of target-response location. Subthreshold depression (StD) demonstrated higher functional activation of left paracentral lobule compared to healthy control (HC). Also, higher functional activation of right putamen was found in StD and major depressive disorder (MDD) compared to HC. (**A**) Clusters of brain functional activation with significant between-group differences (cluster-forming thresholds of P < 0.001 and k ≥ 10). (**B**) Violin plots of z-stats per clusters per group. (**C**) Directed acyclic graph that represents conditional independence associations among depressive symptoms [9 items of Patient-Health Questionnaire-9 (PHQ-9)] and z-stats of 2 brain clusters. Directional association from suicidal ideation to functional activation of left paracentral lobule was found. *PHQ-1* sadness, *PHQ2* anhedonia, *PHQ3* sleep disturbance, *PHQ4* appetite change, *PHQ5* psychomotor change, *PHQ6* fatigue, *PHQ7* self-reproach, *PHQ8* concentration difficulty, *PHQ9* suicidal ideation, *Lt* left, *BA* Brodmann area.

**Figure 4:**
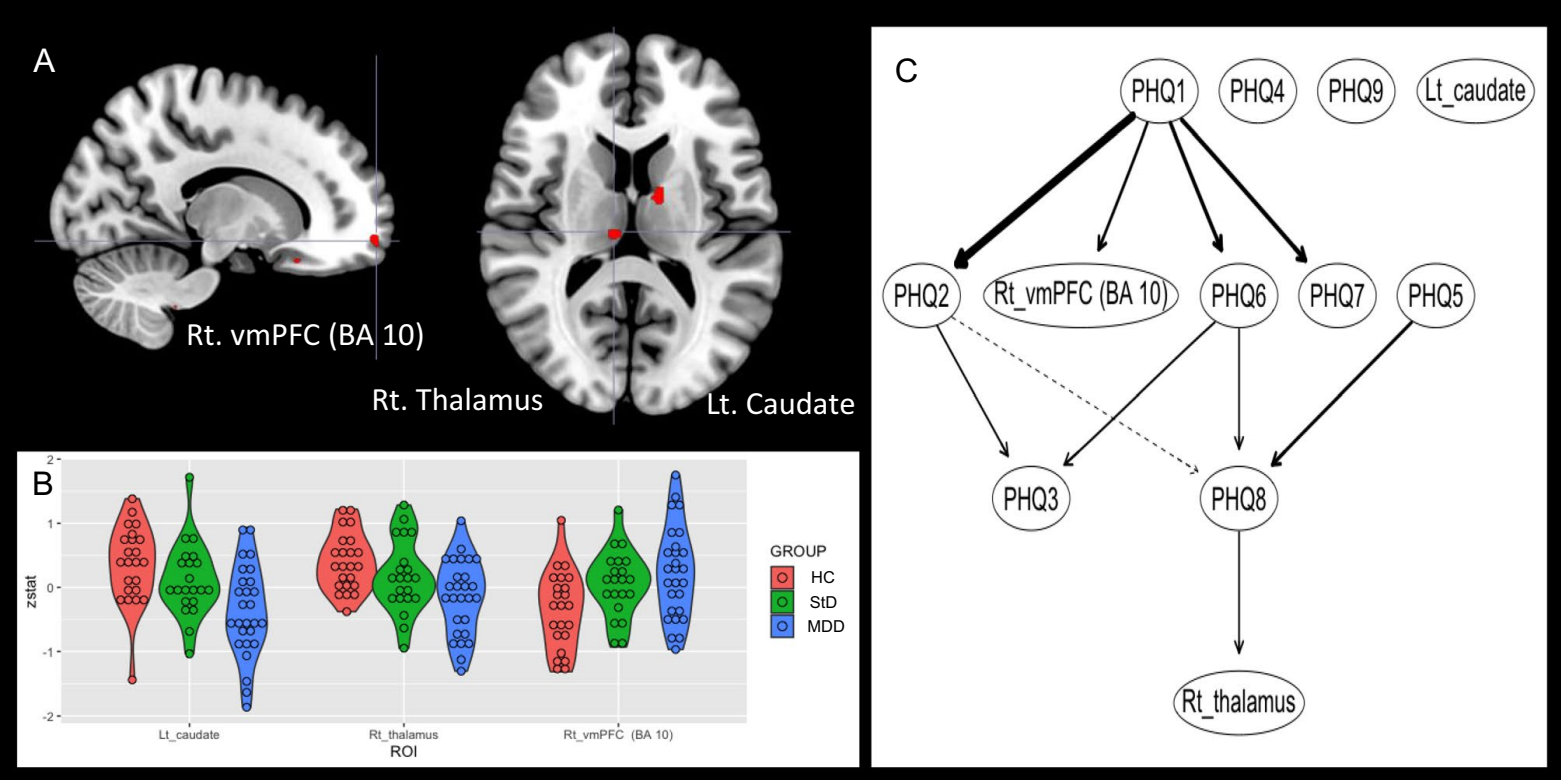
Between-group differences of functional brain activation for conflict control of incongruence trial against larger monetary loss, compared to the smaller monetary loss. Major depressive disorder (MDD) demonstrated higher functional activation of right ventromedial prefrontal cortex, and lower functional activation of left caudate and right thalamus compared to HC. (**A**) Clusters of brain functional activation with significant between-group differences (cluster-forming thresholds of P < 0.001 and k ≥ 10). (**B**) Violin plots of z-stats per clusters per group. (**C**) Directed acyclic graph that represents conditional independence associations among depressive symptoms [9 items of Patient-Health Questionnaire-9 (PHQ-9)] and z-stats of 3 brain clusters. Directional associations from sadness and concentration difficulty to functional activation of ventromedial prefrontal cortex and thalamus were found. *PHQ-1* sadness, *PHQ2* anhedonia, *PHQ3* sleep disturbance, *PHQ4* appetite change, *PHQ5* psychomotor change, *PHQ6* fatigue, *PHQ7* self-reproach, *PHQ8* concentration difficulty, *PHQ9* suicidal ideation, *Lt* left, *BA* Brodmann area.

Temporal derivatives were added to all task regressors to compensate for variability on the hemodynamic response function^65^. Contrasts of interest examined the neural underpinning of conflict control against larger monetary punishment per error response with respect to: (1) conflict control against larger monetary loss, compared to congruence of target-response location (hpci-hpcc; Fig. 2A and Table 2), (2) conflict control against smaller monetary loss, compared to congruence of target response location (lpci-lpcc; Fig. 3A and Table 2), and (3) conflict control of incongruence trial against larger monetary loss, compared to the smaller monetary loss (hpci-lpci; Fig. 4A and Table 2). Overall GLM model was fit for each voxel in the brain using FSL FILM^78^. Moreover, the statistical maps derived from the first-level analysis per fMRI session (and per contrast) were integrated into the statistical maps per participant (and per contrast)—each participants completed a total of 3 fMRI sessions—by fitting the GLM model with a fixed effect modeling implemented in FSL FEAT. Finally, between-group comparisons by way of the voxel-wise analysis of variance [ANOVA; StD (n = 21) vs. HC (N = 27) vs. MDD (n = 23)], independent t tests [StD (n = 21) vs. HC (N = 27); StD (n = 21) vs. MDD (n = 23); HC (n = 27) vs. MDD (n = 23)] in addition to the linear regression of the whole dataset (n = 71) for dependent variable of the PHQ-9 total score were calculated, by fitting the GLM model with a mixed effect model implemented in FSL FEAT with FLAME 1^78^ (FMRIB’s Local Analysis of Mixed Effects)^64^. In these group analyses, age and sex were included as covariates to avoid confounding effects of demographic characteristics. In the current study, no cluster-wise correction was made. Instead, considering the exploratory nature of the study, clusters of functional activations were found from the statistical images by cluster-forming thresholds of P < 0.001 and k (voxel counts) ≥ 10^79^.

### Directed acyclic graph of depressive symptoms and functional brain activation

Further, the current study modeled ‘integrated networks^80^’ of 9 items comprising the PHQ-9 and z-stats of the brain clusters of functional activation (Figs. 2B, 3B, 4B and Table 2) found in the group-level analyses of fMRI data. In other words, directed acyclic graphs (DAGs)^81^ that represent conditional independence relationships or joint probability distribution among depressive symptoms and neural correlates of conflict control against monetary punishment were estimated per contrast [conflict control against larger monetary loss, compared to congruence of target-response location (hpci-hpcc; Fig. 2C), conflict control against smaller monetary loss, compared to congruence of target response location (lpci-lpcc; Fig. 3C), and conflict control against larger monetary loss, compared to the smaller monetary loss (hpci-lpci; Fig. 4C)]. By submitting dataset to a greedy search algorithm named hill-climbing algorithm implemented in an R-package *bnlearn*^82^, a DAG adds edges (connecting different variables or nodes), removes them, and reverses their direction until a goodness-of-fit target score of Bayesian Information Criterion (BIC) is reached^83^. This first step determines whether an edge between two symptoms exists using an iterative procedure of bootstrapping for 10,000 times^83,84^. In the second step, averaged network structure was found by retaining edges with more consistent presence in the 10,000 bootstrapped networks; cut-off point of ‘more consistent presence’ was set by a statistically-driven method retaining edges with high sensitivity and specificity^83,85^. Finally, BIC value was computed for each edge, with higher values (depicted through the edge thickness) signifying higher importance of an edge within the network structure^83^.

### Statistical analyses: clinical and behavioral data

Between-group comparisons of age, years of education, and total scores of self-reports were conducted using ANOVA. Post-hoc comparisons were performed using independent *t*-tests. Comparisons of sex ratio were performed using the chi-squared test. For the behavioral data on task accuracy and response time, a three-way mixed ANOVA with between-subject factors of diagnosis and two within-subject factors, including the sizes of monetary loss and congruency of target-response location, was conducted using IBM SPSS Statistics version 23 (https://www.ibm.com). Note that the trials with correct but delayed responses were included in analyses of responses time data because the research interest was not limited on the behaviors resulting in successful avoidance of monetary loss but did involve the behaviors for the avoidance. Additionally, for the analyses of response time (RT), the trials with incorrect response were excluded. The threshold of statistical significance was set at P < 0.05 (Bonferroni-corrected).

## Results

### Demographic and clinical information

Table 1 summarizes the demographic and clinical information (total scores or sub-item scores of self-reports) of individuals who participated in the current study. Significant differences in age, sex ratio, and years of education were not observed between the MDD, StD, and HC (mean ± standard deviation [SD] for n = 71; age = 24.5 ± 2.9 years; years of education = 16.7 ± 2.3 years; male-to-female ratio = 38:33). Conversely, significant between-group differences were observed for the intensity of depressive mood and levels of generalized anxiety, state anxiety, self-esteem, positive self-appraisal for coping skills, perceived social support, life satisfaction, and hopelessness (all P < 0.05/11 [number of clinical information scores] = 0.005). Post-hoc analyses also showed significant differences between the MDD and HC (in all eight clinical information scores listed above) and between the StD and HC (for all clinical information listed above other than anxiety [GAD-7] and perceived social support), but not between the MDD and StD (defined significant for all P < 0.005).

**Table 1:** Demographic and clinical characteristics.

Variables	HC (n = 23)		StD (n = 21)		MDD (n = 27)		F / Chi^2	P value	Post-hoc: HC vs. StD			post-hoc: HC vs. MDD			Post-hoc: StD vs. MDD		
	M	SD	M	SD	M	SD			T	df	P value	T	df	P value	T	df	P value
Age	24.65	2.87	24.33	3.04	24.59	2.80	0.075	0.9278	*NA*								
Sex (M/F)	13	10	12	9	13	14	0.51	0.7760	*NA*								
Education years	16.57	2.13	16.46	2.02	16.96	2.64	0.33	0.7205	*NA*								
PHQ-9 total	3.17	2.55	8.29	4.05	12.04	3.92	38.17	< 0.001*	− 4.95	33	< 0.001*	− 9.29	48	< 0.001*	− 3.24	46	0.0022*
GAD-7 total	2.30	2.34	5.10	4.07	8.74	4.96	18.79	< 0.001*	− 2.75	31	0.0098	− 6.00	38	< 0.001*	− 2.73	46	0.0090
STAI-S total	42.09	9.04	52.29	7.85	57.89	7.91	22.92	< 0.001*	− 3.98	42	0.0003*	− 6.59	48	< 0.001*	− 2.44	46	0.0185
RSES total	30.52	3.89	26.14	4.59	23.89	4.43	14.94	< 0.001*	3.42	42	0.0014*	5.57	48	< 0.001*	1.72	46	0.0918
RAS total	44.52	7.83	37.10	6.05	34.48	9.13	10.50	< 0.001*	3.50	42	0.0011*	4.13	48	0.0001*	1.13	46	0.2638
Social support total	95.57	11.03	86.76	17.38	78.67	17.64	7.18	0.0015*	2.02	42	0.0493	4.12	44	0.0002*	1.59	46	0.1193
**QOL total**	82.52	12.39	68.90	12.07	63.26	12.19	15.94	< 0.001*	3.69	42	0.0006*	5.53	48	< 0.001*	1.60	46	0.1169
QOL: physical	25.39	4.71	20.76	4.84	19.52	4.12	11.20	0.0001*	3.22	42	0.0025*	4.71	48	< 0.001*	0.96	46	0.3413
QOL: psychological	19.96	4.04	14.76	3.40	12.63	3.64	25.07	< 0.001*	4.59	42	< 0.001*	6.75	48	< 0.001*	2.07	46	0.0440
QOL: social	10.48	1.95	9.43	2.44	8.00	2.29	7.78	0.0009*	1.58	42	0.1211	4.08	48	0.0002*	2.08	46	0.0427
QOL: environmental	26.70	4.54	23.95	5.75	23.11	4.68	3.42	0.0384	1.76	42	0.0848	2.74	48	0.0086	0.56	46	0.5787
**BIS total**	64.30	10.00	69.33	12.62	69.69	12.65	1.52	0.2264	*NA*								
BIS: attentional	17.43	4.25	20.62	4.02	20.04	4.31	3.70	0.0299	− 2.55	42	0.0146	− 2.14	48	0.0374	0.48	46	0.6350
BIS: motoric	20.26	4.13	22.29	5.17	22.94	5.00	2.06	0.1356	*NA*								
BIS: non-planning	26.61	4.49	26.43	5.55	26.70	5.24	0.02	0.9828	*NA*								
BHS total	4.09	3.85	8.76	5.23	8.98	4.82	8.25	0.0006*	− 3.40	42	0.0015*	− 3.92	48	0.0003*	− 0.15	46	0.8808

*BHS* Beck hopelessness scale, *BIS* Barratt impulsiveness scale, *GAD-7* generalized anxiety disorder-7, *HC* healthy controls, *MDD* major depressive disorder, *M* mean, *MDS* mild depressive symptoms, *PHQ-9* patient health questionnaire-9, *QOL* World Health Organization quality of life abbreviated version, *RAS* resilience appraisal scale, *RSES* Rosenberg self-esteem scale, *SD* standard deviation, *STAI-S* state and trait anxiety inventory-state anxiety scale.

*P < 0.05/11 [number of clinical information scores] = 0.005.

### Performance of color-space conflict control against the punishment of monetary loss

Possible effects of the size of monetary loss, congruency, and diagnosis on the mean reaction time of correct trials for each participant (Fig. 1B) were also examined using three-way mixed ANOVA. The results did not show a significant three-way interaction effect on reaction time [F (2,68) = 1.223, P = 0.301]. Conversely, a statistically significant simple two-way interaction between the size of monetary loss versus congruency was found in MDD [F (1, 26) = 35.4, P = 2.78 × 10^–6^] but not in the HC (P = 0.724) or StD (P = 0.638). In MDD, compared with a smaller monetary loss, knowing the penalty of a larger monetary loss resulted in a faster response for a congruent target-response location [mean ± SD = 0.544 ± 0.091 s (smaller monetary loss), 0.528 ± 0.085 s (larger monetary loss); F (1,26) = 13.2, P = 0.001] but not for an incongruent location [0.570 ± 0.093 s (smaller monetary loss), 0.565 ± 0.087 s (larger monetary loss); F (1,26) = 1.04, P = 0.317]. In terms of simple main effects, a statistically significant difference in reaction time was only found when there was a conflict in target-response location [F (1, 68) = 69.926, P = 4.82 × 10^–12^] but not for the size of monetary loss (P = 0.301) or diagnosis (P = 0.985).

Regarding the accuracy of task performance (Fig. 1C), three-way mixed ANOVA did not show a statistically significant three-way interaction between the size of monetary loss, congruency, and diagnosis (P = 0.761). Similarly, statistically significant simple two-way interactions were not observed between the size of monetary loss and congruency in MDD (P = 0.699), StD (P = 0.666), or HC (P = 0.596). Regarding main effects, a statistically significant difference in accuracy was found for conflict in target-response location [F (1, 68) = 14.616, P = 2.88 × 10^–4^] but not for the size of monetary loss (P = 0.056) or diagnosis (P = 0.830).

### fMRI analyses & directed acyclic graph of depressive symptoms and functional brain activation

Group-level GLM analyses of fMRI data illustrated possible neural correlates of conflict control against larger monetary punishment per error response with respect to: (1) conflict control against larger monetary loss, compared to congruence of target-response location (hpci-hpcc), (2) conflict control against smaller monetary loss, compared to congruence of target response location (lpci-lpcc), and (3) conflict control of incongruence trial against larger monetary loss, compared to the smaller monetary loss (hpci-lpci; Fig. 4A and Table 2). First, conflict control against larger monetary loss (Fig. 2 and Table 2) was associated with higher functional activation of left insula [Brodmann area (BA) 13] in StD than HC [MNI coordinates (x,y,z) = − 48 0 − 2; z = 3.37, P (uncorrected) = 3.72 × 10^–4^, k (cluster size) = 10], and MDD [MNI coordinates (x,y,z) =  − 46 4 − 6; z = 3.48, P = 2.46 × 10^–4^, k = 10]. StD also showed lower functional activation of left dorsal anterior cingulate [dACC (BA 32); MNI coordinates (x,y,z) =  − 8 40 12; z = 3.93, P = 4.24 × 10^–5^, k = 11] compared to MDD. Higher functional activation of left precuneus [BA 31; MNI coordinates (x,y,z) =  − 10 − 70 30; z = 3.74, P = 9.19 × 10^–5^, k = 16] in MDD compared to HC was also shown. On the other hand, clusters of functional activations were not found from the ANOVA [StD (n = 21) vs. HC (N = 27) vs. MDD (n = 23)] nor from the linear regression of the whole dataset (n = 71) for dependent variable of the PHQ-9 total score [cluster-forming thresholds of P < 0.001 and k (voxel counts) ≥ 10]. In ‘depressive symptom-brain activation’ network of DAG, degree of functional activation in left insula was dependent on functional activation of left dACC and was not dependent on any item-level depressive symptoms (Fig. 2C and Table 2).

**Table 2:** Between-group comparisons for the task-related brain activation: control of color-location conflict in the middle of monetary punishment for error or delayed responses.

CONTRAST	Statistical analyses	Peak Z	Peak P	df	Cluster size	MNI coordinates			Laterality	BA	Brain regions	ROI_network analyses_zstst**
						x	y	z				
Conflict control against larger monetary loss, compared to congruence of target-response (hpci-hpcc)	HC < StD	3.37	0.000372	40	10	− 48	0	− 2	Left	BA 13	Insula	[2]
	MDD > StD	3.93	4.24E−05	44	11	− 8	40	12	Left	BA 32	Dorsal anterior cingulate cortex	1
	MDD < StD	3.48	0.000246		10	− 46	4	− 6	Left	BA 13	Insula	2
	HC < MDD	3.74	9.19E−05	46	16	− 10	− 70	30	Left	BA 31	Precuneus	3
Conflict control against smaller monetary loss, compared to congruence of target-response (lpci-lpcc)	HC < StD	3.99	3.29E−05	40	14	28	4	− 8	Right	–	Putamen	4
		3.88	5.14E−05		11	− 12	− 34	54	Left	BA 5	Paracentral lobule	5
	HC < MDD	3.7	0.000108	46	11	28	4	− 8	Right	–	Putamen	[4]
Conflict control against larger monetary loss, compared to smaller monetary loss (hpci-lpci)	HC > MDD	4.04	2.71E−05	46	21	− 12	− 4	14	Left	–	Caudate body	6
		3.71	0.000103		10	8	− 20	12	Right	–	Medial dorsal nucleus of thalamus	7
	HC < MDD	3.81	7.07E−05		18	14	64	− 10	Right	BA 10	Ventromedial prefrontal cortex	8

Cluster-forming thresholds were p < 0.001 and k (voxel number) ≥ 10.

*BA* Brodmann area, *HC* healthy controls, *MDD* major depressive disorder, *StD* subthreshold depression, *hpci* incongruent trial against larger monetary loss, *hpcc* Congruent trial against larger monetary loss, *lpci* incongruent trial against smaller monetary loss, *lpcc* Congruent trial against smaller monetary loss.

Second, for conflict control against smaller monetary loss (Fig. 3 and Table 2), higher functional activation of left paracentral lobule [BA 5; MNI coordinates (x,y,z) =  − 12 -34 54; z = 3.88, P = 5.14 × 10^–5^, k = 11] was found in StD compared to HC. Also, higher functional activation of right putamen [MNI coordinates (x,y,z) = 28 4 − 8] was shown in StD [z = 3.99, P = 3.29 × 10^–5^, k = 14] and MDD [z = 3.70, P = 1.08 × 10^–4^, k = 11] compared to HC. Clusters of functional activations were not found from the ANOVA [StD (n = 21) vs. HC (N = 27) vs. MDD (n = 23)] nor from the linear regression of the whole dataset (n = 71) for dependent variable of the PHQ-9 total score. In DAG, directional association from suicidal ideation (PHQ9) to the functional activation in left paracentral lobule was found (Fig. 3C and Table 2). Third, for conflict control against larger monetary loss compared to the smaller monetary loss, MDD revealed higher functional activation of right ventromedial prefrontal cortex [vmPFC (BA 10); MNI coordinates (x,y,z) = 14 64 − 10; z = 3.81, P = 7.07 × 10^–5^, k = 18], and lower functional activation of left caudate body [MNI coordinates (x,y,z) =  − 12 − 4 14; z = 4.04, P = 2.71 × 10^–5^, k = 21] and right medial dorsal nucleus of thalamus [MNI coordinates (x,y,z) = 8 − 20 12; z = 3.71, P = 1.03 × 10^–4^, k = 10] compared to HC. Clusters of functional activations were not found from the ANOVA [StD (n = 21) vs. HC (N = 27) vs. MDD (n = 23)] nor from the linear regression of the whole dataset (n = 71) for dependent variable of the PHQ-9 total score. DAG demonstrated directional associations from sadness (PHQ1) and concentration difficulty (PHQ8) to vmPFC and thalamus, respectively (Fig. 4C and Table 2).

## Discussion

In the current study, between-group differences of functional brain activation for the conflict control in the middle of monetary punishment among the MDD, StD, and HC were examined. During performance of conflict control, incongruence of target-response location resulted in slower reaction time (Fig. 1B) and lower response accuracy (Fig. 1C) compared to congruent condition regardless of the size of monetary punishment and diagnosis. Conflict control against larger monetary loss was associated with higher functional activation of left insula (BA 13) in StD than HC and MDD. StD also showed lower functional activation of left dACC (BA 32) compared to MDD for conflict control against larger monetary loss (Fig. 2A and Table 2). In ‘depressive symptom-brain activation’ network of DAG, degree of functional activation in left insula was dependent on functional activation of left dACC and was not dependent on any item-level depressive symptoms (Fig. 2C). For conflict control against smaller monetary loss, StD demonstrated higher functional activation of left paracentral lobule (BA 5) and right putamen compared to HC (Fig. 3A and Table 2). For conflict control against larger monetary loss compared to the smaller monetary loss, MDD revealed higher functional activation of right vmPFC (BA 10), in addition to the lower functional activation of left caudate and right medial dorsal nucleus of thalamus compared to HC (Fig. 4A and Table 2). DAGs showed directional associations from suicidal ideation, sadness, and concentration difficulty to functional activation of paracentral lobule, ventromedial prefrontal cortex, and thalamus for conflict control against monetary loss (Figs. 3C and 4C).

In the current study, functional activation of left insula for conflict control against larger monetary loss was higher in StD compared to HC and MDD. Moreover, StD also showed higher functional activation of left dACC for conflict control against larger monetary loss than MDD. In DAG (Fig. 2C), functional activation of dACC could predict functional activation of left insula for conflict control against larger monetary loss. In consideration of the recent studies that illustrated differential functional connectivity strengths of StD between insula versus amygdala^18^ and superior frontal cortex^86^ for emotional regulation tasks compared to HC, the current study result could partly be a reflect of emotional regulation against monetary punishment for error responses in StD. Neural correlates of conflict control and response inhibition in the Simon spatial incompatibility task has been attributed to the regions comprising the cingulo-opercular and default-mode networks including the anterior–posterior cingulate and anterior–posterior insula^87^. Of note, insula and cingulate (in addition to the striatum, amygdala, and thalamus) are associated with the anticipation and processing of possible monetary loss^88^. Further, uncertainty in the monetary outcome is associated with functional activation of the anterior insula, dorsal anterior cingulate, and the nucleus accumbens for male HC^89^. On the contrary, MDD demonstrates selective functional activation of the anterior insula only in response to social stimuli (both acceptance and rejection) but not monetary stimuli^90^. Also, MDD with a suicidal attempt history exhibited greater aversion to a risk of monetary loss and had attenuated functional activation of the amygdala and insula compared with HC^91^. In adolescence, stronger functional connectivity between insula and orbitofrontal cortex for stimuli of monetary loss could predict depressive symptoms at 9-month follow-up^92^. As functional activation of the anterior insula for monetary punishment undergoes a quadratic pattern of development comprised of temporary reduction during mid-late adolescence and upward increment at early adulthood^93^, suffer of depressive symptoms during young adulthood could affect the pattern of brain functional activation of insula in response to monetary loss.

In consideration of possibility that associations between depressive symptom and functional brain activations versus could vary for different dimensions of depressive symptoms^36^, we modeled ‘integrated networks^80^’ of conditional independence relationships^81,83,84^ among the item-level depressive symptom of PHQ-9^37,38^ and z-stats of functional activation clusters for conflict control against monetary punishment. First, for conflict control against smaller monetary loss (Fig. 3A,B and Table 2), directional association from suicidal ideation (PHQ9) to the functional activation in left paracentral lobule (higher functional activation of StD than HC found in the GLM analyses) was shown in DAG (Fig. 3C). Both StD and MDD are associated with increased prevalence of lifetime suicidal attempt^94^. In addition, neuroimaging studies have reported stronger paracentral lobule-amygdala resting-state functional connectivity in mood disorder with suicidal behavior^95^ and association between the cortical thickness value of paracentral lobule versus non-planning impulsivity in MDD^96^. Therefore, higher functional activation of paracentral lobule for conflict control against smaller monetary punishment could be considered as a neural correlates of suicidal ideation.

The current study have some limitations. First, the study design was cross-sectional, and temporal consistency in the study results should be examined using additional studies with a longitudinal follow-up design. Second, to enhance the statistical power of the study results, future studies with a larger sample size are necessary. Third, no cluster-wise correction has been made. Instead, considering the exploratory nature of the study, clusters of functional activations were found from the statistical images by cluster-forming thresholds of P < 0.001 and k (voxel counts) ≥ 10 for the fMRI data analyses^79^. Results of the current study has to be considered preliminary and are in need of replication in future studies.

## Conclusion

StD showed higher functional activation of left insula for conflict control against larger monetary loss compared to HC and MDD. StD also showed higher functional activation of left dACC for conflict control against larger monetary loss than MDD. Functional activation of dACC could predict functional activation of left insula for conflict control against larger monetary loss. Differential functional activation of salience network components in StD for conflict control against monetary punishment could be a brain phenotype of subthreshold depression. Item-level depressive symptoms of suicidal ideation, sadness, and concentration difficulty could be reflected in the conflict control-related functional activation of paracentral lobule (against smaller monetary loss), vmPFC and thalamus (against larger monetary loss), respectively.

Further studies that examine the possible utility of the functional activation patterns at insula and dACC in StD for predicting the short-term and long-term prognosis of depressive symptoms (*i.e.,* recovery to non-depressive status vs. conversion to MDD) are warranted.
